# Structural Equation Modeling of a Global Stress Index in Healthy Soldiers

**DOI:** 10.3390/jcm10081799

**Published:** 2021-04-20

**Authors:** Tanja Maier, Melanie Kugelmann, Dae-Sup Rhee, Sebastian Brill, Harald Gündel, Benedikt Friemert, Horst-Peter Becker, Christiane Waller, Manuela Rappel

**Affiliations:** 1 Clinic for Psychosomatics and Psychotherapeutic Medicine, Ulm University Medical Center, 89081 Ulm, Germany; melanie.kugelmann@gmx.de (M.K.); dae-sup.rhee@uni-ulm.de (D.-S.R.); Harald.Guendel@uniklinik-ulm.de (H.G.); christiane.waller@klinikum-nuernberg.de (C.W.); manuela.rappel@uni-ulm.de (M.R.); 2 Military Hospital, 89081 Ulm, Germany; sebastian.brill@web.de (S.B.); bendiktfriemert@bundeswehr.org (B.F.); 3 Military Hospital, 10115 Berlin, Germany; HorstPeterBecker@bundeswehr.org; 4 Department of Psychosomatics and Psychotherapeutic Medicine, Paracelsus Medical University of Nueremberg, 90419 Nueremberg, Germany

**Keywords:** psychosocial stress, structural equation model, cardiovascular disease, risk factors, global stress index

## Abstract

Accumulation of stress is a prognostic trigger for cardiovascular disease. Classical scores for cardiovascular risk estimation typically do not consider psychosocial stress. The aim of this study was to develop a global stress index (GSI) from healthy participants by combining individual measures of acute and chronic stress from childhood to adult life. One-hundred and ninety-two female and male soldiers completed the Perceived Stress Scale (PSS4), Trier Inventory for Chronic Stress (TICS), Hospital Anxiety and Depression Scale (HADS), Childhood Trauma Questionnaire (CTQ), Posttraumatic Diagnostic Scale Checklist (PDS), and the Deployment Risk and Resilience Inventory (DRRI-2). The underlying structure for the GSI was examined through structural equation modeling. The final hierarchical multilevel model revealed fair fit by taking modification indices into account. The highest order had a g-factor called the GSI. On a second level the latent variables stress, HADS and CTQ were directly loading on the GSI. A third level with the six CTQ subscales was implemented. On the lowest hierarchical level all manifest variables and the DRRI-2/PDS sum scores were located. The presented GSI serves as a valuable and individual stress profile for soldiers and could potentially complement classical cardiovascular risk factors.

## 1. Introduction

Psychosocial stress is a common term in everyday life and was originally defined as a nonspecific reaction of the body to a noxious environmental stimulus [1]. Lazarus described a more psychological approach by analysing appraisal and coping mechanisms [2]. Over the years, the importance of stress for psychological and physical health and well-being has been widely accepted and stress-related research has increased considerably [3,4]. Psychosocial stress has been found to be a cardiovascular (CV) risk factor [5] for physically healthy individuals and patients with manifest diseases [6,7] and has been shown to be directly linked to the incidence of myocardial infarction (MI) [8]. Additionally, psychosocial stress is closely linked to depression and traumatic experiences, as well as specific life events and private/occupational burden [9,10,11].

Depressive episodes have been explained as a maladaptive phenomenon due to chronic stress or environmental changes [12]. Depressive and anxiety disorders were significantly associated with increased CV incidents in a 6-year follow up [13]. Higher odds ratios were also shown for depressive symptoms measured with the Hospital Anxiety and Depression Scale (HADS) in predicting incident cardiovascular disease (CVD) [14]. Stress and stressful live events showed robust causal associations to depression [10] and anxiety [15]. Kendler and Gardner [16] combined recent stressful live events within the last month like job loss, marital problems, or death and found associations to depressive vulnerability and depression. Work stress acts as an independent risk factor for depression and anxiety, especially high job demands like time pressure [17,18] and is associated with CVD risk factors like triglycerides, cholesterol, smoking, and systolic blood pressure [19]. Furthermore, critical events over the lifespan have an impact on experienced psychosocial stress levels: major negative life events in adulthood like divorce or death of a loved one led to a significant increase in daily life stress [20] and the accumulation of major life events showed a dose-response relationship with the risk of stroke [21]. Among depression and negative life events, psychosocial stress also implies experiences of acute and chronic stress: daily hassles like work interruptions which causes an increase in experienced stress had a mediating impact on major life events and psychological symptomatology [22]. Sources of self-reported chronic stress can be interpersonal difficulties, (work) overload or goal-striving stress [23], and in a 21-year follow-up it was found that it significantly increases the risk of stroke, coronary heart disease (CHD), or CV events [24]. However, not only events during adulthood showed associations, in individuals with experiences of child maltreatment (CM) manifestations in the biological stress response was found—e.g., higher and prolonged levels of cortisol after social stress induced by the Trier Social Stress Test compared to individuals without CM-experiences [25]. Neuroendocrine changes in the stress response were linked to CM and current stress was a mediator of CM and depression [26]. Additionally, CM served as a predictor of increased psychological stress levels in adulthood [27] and showed a strong graded relationship with leading causes of death like smoking, obesity, depression, or CHD in adults [28] with a marked dose–response impact on CVD [29]. Especially military personnel have a higher risk for perceived job-stress than civilians [30]. They face high rates of cardiovascular disease and deployed soldiers are more likely to suffer from hypertension due to increased mental stress than non-deployed soldiers [31,32].

Classical risk factor scores consisting of gender, age, smoking, blood pressure, family history of MI, lipoproteins, and diabetes mellitus have been widely used for the calculation of the 10-year risk of a fatal CV disease event [33,34,35]. It is considered as a simple and accurate way for predicting MI in clinical practice [36]. New factors have emerged in recent years that can also trigger MI or predict CV risk precisely. Yusuf et al. [8] showed that regular consumption of fruits/vegetables, moderate alcohol consumption and moderate physical exercise are protective and associated with MI risk reduction, whereas psychosocial factors have an additional influence on the risk of MI in both sexes and at all ages. Multivariate analyses revealed that smoking and raised ApoB/ApoA1 lipid ratio—followed by history of diabetes, hypertension, and psychosocial stress—measured via positive exposure to depression, perceived stress at home or work, low locus of control and major life events were the strongest risk factors. High perceived mental stress was linked to a 1.5-fold increased CHD risk compared to low stress levels [37,38]. For instance, patients with an acute MI and high acute stress (PSS4), showed increased 2-year mortality [39].

The approach to define one global stress index for soldiers needs to consider a multidimensional conceptualization of stress. To potentially use this index in further research as a cumulated stress trigger for CVD events, the structure was developed following Yusuf et al. [8]. However, research that analyses the underlying structure and not only the construct level of psychosocial stress is lacking. It is not fully understood how the items between different stress-based questionnaires are linked to each other.

Therefore, the aim of the study was to develop a latent ‘global stress index’ (GSI) based on the aspects of acute and chronic perceived stress, depressive, and anxiety symptoms, stressful life events and CM as part of the BEST study (German armed forces deployment and stress) on healthy male and female German soldiers.

## 2. Materials and Methods

### 2.1. Study Design and Recruitment

The analysis was performed on data from the BEST study. The prospective design aimed to investigate bio-psycho-social stress effects of foreign deployment on CV health in German Armed Forces. Therefore, soldiers were asked to participate at three different time points prior to (t0), 4–6 weeks after (t1) and one year after (t2) mission abroad. The control group with similar age and gender distribution participated at the given time points without mission abroad. Both groups performed consecutive social stress tests using the Trier Social Stress Test for Groups (TSST-G [40]) and psychological as well as biological measures at baseline and during the stress task. This paper is based on the psychological baseline data prior to foreign deployment for both groups. Soldiers were recruited from German military barracks in Dornstadt, Laupheim, and Ulm as well as in the German Armed Forces hospital in Ulm, Germany.

### 2.2. Participants and Missing Values

In the study t0 was completed by 234 participating soldiers. Due to missing questionnaires 42 soldiers were excluded as a result of a study recess. In the SEM analysis missing data were estimated using the full-information maximum likelihood (FIML) method (Enders & Bandalos, 2001) considering all information of the observed data like mean and variance. In total, data of 192 male and female soldiers were used for further calculations. The mean age of the soldiers was *M* = 30.07 *SD* = 6.88 (range 19–56) years. 27.1% of the achieved participants were females.

### 2.3. Measures

To measure different aspects of psychosocial stress the following questionnaires were used. Chronic perception of stress in the last three months was covered by the Trier Inventory for Chronic Stress (TICS [41]) and acute stress in the last month by the Perceived Stress Scale (PSS4 [42]). The scales included questions to work-related stress, social stressors and daily hassles (e.g., high expectations, disputes and tension, lack of respect). In the PSS4 item 1 and 4 are phrased as negative questions. The Posttraumatic Stress Diagnostic Scale Checklist (PDS [43]) quantified potential traumatizing events (e.g., violence, sexual abuse, natural disasters) over lifespan. The Childhood Trauma Questionnaire (CTQ [44]) quantified childhood maltreatment and consisted of six subscales which were emotional abuse (EA), physical abuse (PA), sexual abuse (SA), emotional neglect (EN), physical neglect (PN), and a scale with minimization questions (MQ). The section “prior stressors” from the Deployment Risk and Resilience Inventory (DRRI-2 [45]) asked for highly stressful live events (e.g., divorce, financial problems, death of related persons). The two subscales of the Hospital Anxiety and Depression Scale (HADS [46]) measured symptoms of anxiety and depression (e.g., worrying about the future, loss of joy, feeling anxious). All instruments were validated. Due to the binary item structure (1 = occurrence vs. 0 = non-occurrence) a total sum score for all items of the PDS and DRRI-2 was used. For PSS4, TICS, HADS, and CTQ all single items were considered in the SEM. Reverse coded items were inverted prior to the analysis with higher values indicating more negative outcomes. All item statistics are described below (Table A1).

### 2.4. Data Analysis

Data were edited using open-source software R [47]. The software packages foreign [48], psych [49], lavaan [50], and nvnormtest [51] provided additional functionality. Graphics were generated with AMOS [52]. In advance, calculations of means, standard deviations and Shapiro–Wilk tests for item distribution were performed. The Pearson correlation matrix was used to show variances between items. To examine multivariate normality in the whole item set the Mardia and Henze-Zirkler test for multivariate normality were conducted. The structural equation model was used to analyze the relationships between all stress-related items. Within R all intercepts in the SEM were set to zero by default. A two-step modeling approach was adopted. First, the structure of all variables was modified through creating latent variables based on the measured variables. The validated structure of questionnaires served as a basis for initial iterations of calculating the GSI. Comparing the fit of unnested models in SEM the Akaikes and Bayesian Information Criteria (AIC/BIC [53,54]) were taken into account. For this comparison lower values indicated better model fit. Calculating *Χ²* tests allowed verifying the significance of improvement within nested models. The root-mean-square errors of approximation (RMSEA) provided additional information about the fit within all nested models. Referring to several authors [55,56] values above 0.10 show poor model fit, values below 0.10 suggest mediocre fit, values below 0.08 indicate fair fit and values below 0.05 are a sign for close fit. The smaller the value the better the fit. We aimed to reach at least a fair fitting model. Second, relationships among the variables on different levels were tested exploratory to get an adequate fit. Modification indices were calculated. They indicate which links between variable can improve the model fit. Adequate RMSEA fit was reached by iteratively observing increased modification indices after every change. Step by step the highest values that could be theoretically justified were implemented in the model until adequate RMSEA, fair fit, was reached.

## 3. Results

### 3.1. Descriptive and Initial Data Analyses

Mean, minimum, maximum and standard deviation of all items are presented in Table A1. A very low standard deviation was found for item CTQ_21: “Someone threatened to hurt me or tell lies about me unless I did something sexual with them” (*SD* = 0.39), accompanied by a relatively low mean (*M* = 1.05). None of the items achieved normal distribution, the Shapiro–Wilk test was significant (*p* < 0.001) over all items (Table A1). Similarly, the multivariate normal distribution was not fully given for the whole item set, except for the skewness in Mardia test (HZ = 464, *p* < 0.001; skewness: *Χ*² = 185785, *p* = 1.00; kurtosis: z = −17.84, *p* < 0.001). The Pearson correlation coefficients measured for all items ranged between approximately zero (e.g., r = 0.006 PDS and tics18) and r = 0.75 (ctq28 and ctq19). There were also some highly negative correlations as r = −0.79 (ctq28 and ctq22). Within the items of the same questionnaire the correlations were expectedly higher than between items of different questionnaires (correlation tables are available on request from the corresponding author).

### 3.2. Structural Equation Model (SEM)

**Figure 1:**
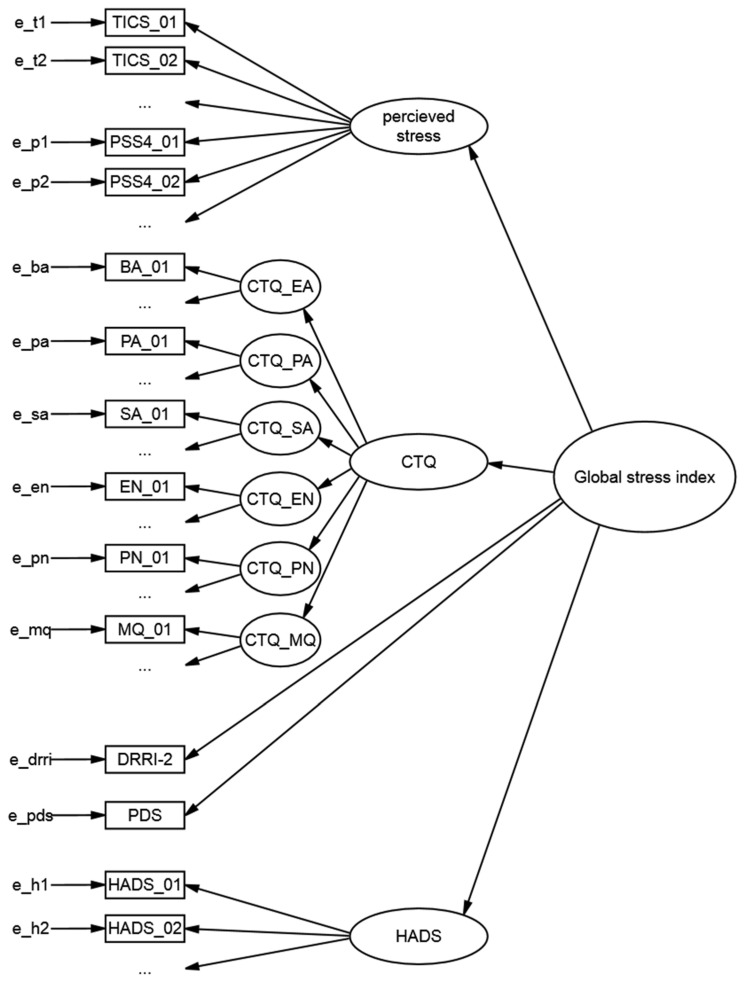
Illustration of the multilevel model results from structural equation modeling. All manifest variables are presented in squares. All latent factors are presented in circles. e = error; PSS4 = Perceived Stress Scale; TICS = Trier Inventory for Chronic Stress; HADS = Hospital Anxiety and Depression Scale; PDS = Posttraumatic Stress Diagnostic Scale Checklist; DRRI_2 = Deployment Risk and Resilience Inventory; CTQ = Childhood Trauma Questionnaire; EA = Emotional Abuse; PA = Physical Abuse; SA = Sexual Abuse; EN = Emotional Neglect; PN = Physical Neglect; MQ = minimization questions.

The next step in the calibration of a GSI model was to perform SEMs. The presumed model structure for the SEM was influenced by the given scales and subscales of the questionnaires that had already been validated. An overview of the statistics and fit values of all models is given in Table 1 At first a g-factor model was established with all items loading on the GSI factor. As it is shown in Table 1 the RMSEA of 0.108 indicated poor model fit. All items showed significant loadings on the general factor. It was consistent throughout all models 1–17 that all variable loadings were highly significant (*p* < 0.001). As a next step (model 2) all items belonging to one questionnaire were summarized to one latent factor. For example, all 12 HADS items were loading on one latent HADS factor. Furthermore, all latent questionnaire factors and the DRRI-2/PDS sum scores were directly loading on the GSI factor. For model 2, no valid results could be calculated, some estimated variances were negative. In model 2a, the latent variables TICS and PSS4 were loading on a stress factor of higher order but this approach was not identified. As this also did not lead to a proper solution model 3 was proposed similar to model 2 with the difference that all TICS and PSS4 items were directly combined to one factor labeled perceived stress. AIC and BIC proved model 3 to be superior to model 1. RMSEA was improved to 0.100. Consequently, the higher order factor perceived stress was retained in the following models. Differentiating HADS into its subscales deteriorated the AIC/BIC (model 4, Table 1). Combining the two HADS subscales to a higher order factor in model 4a led to an unidentified model. Based on model 3 differentiating CTQ into its subscales improved model fit (AIC/BIC). RMSEA in model 5 indicated mediocre fit of 0.091. Therefore, the subdivision was also part of subsequent models. The last model based on theoretical considerations was model 6, all CTQ subscales were combined to one CTQ higher order factor (Figure 1). Model 6 was superior to model 5 comparing the AICs and BICs. Again, a mediocre fit was achieved (RMSEA = 0.091).

As the theoretically based, deductive method did not lead to a satisfactory model fit, the model was further improved by taking modification indices into account until a fair fit was reached (see Table 1). First, a critical item (CTQ_21) was identified and removed from the dataset. Modification indices suggested that the item was not represented adequately by the assumed factor. Many cross loadings would be required indicating that the item was not measuring the proposed construct. Prior analyses already revealed that item CTQ_21 had the lowest standard deviation. Considering all evidence, the item did not provide enough information and was therefore excluded from model 7. The following models were all nested. X² tests revealed that all improvements were significant (Table 1). Modification indices suggested improving the fit through implementing an intercept to the GSI factor (model 8) followed by adding it to CTQ_MQ, PSS4_02 and PSS4_03 (model 9). Adding the link between two scales of higher order (CTQ_EN and CTQ_MQ) to model 10 also improved the RMSEA significantly. The expectable negative correlation between emotional neglect “I didn’t feel loved” and minimization questions like “There was nothing I wanted to change about my family” highlighted the content related relation of the family background. Including correlations of mainly TICS items led to better fit in the following models. The highest values of modification indices suggested cross loadings of TICS_10 (“I’m missing interesting tasks which fill the day”) with semantically closely linked items like “There are times where I don’t do anything meaningful” (models 11,12). In model 13–16 other item correlations were added to the SEM on condition that high modification indices were detected and the combinations were reasonable in the way of a content-related fit (Table 1). In a last step the high effect between CTQ_SA and item CTQ_25 was considered as suggested in the statistic of modification indices. This led to the final model (model 17) with a fair fit of 0.079, so there was no need for further modifications. All parameter statistics and a figure of the overall model are shown in Table A2 and Figure 1. This version of the structural equation modeling analysis indicated that the final model 17 fit the underlying data well.

## 4. Discussion

The aim of the study was to develop the GSI for soldiers based on different stress-related questionnaires and to reveal the underlying item structure. This was reached by successfully establishing a structural equation model measuring the GSI. The final hierarchical multilevel model revealed fair fit. On the highest order was a g-factor called the “GSI”, the newly developed global stress index. On a second level the following latent variables were directly loading on the GSI: perceived stress, HADS [46] and CTQ [44]. The variable perceived stress was directly measured by the combination of all TICS [41] and PSS4 [42] items, which means that the items of these questionnaires are closer linked than with the remaining questionnaires and consequently covered by a higher-order factor. Compared to that, the CTQ was divided into its six subscales. Therefore, a third level was implemented containing the underlying constructs EA, PA, SA, EN, PN, and MQ which seemed to have an additional informative influence on the GSI. All manifest variables were located on the lowest hierarchical level. This level also encompassed the DRRI-2 [45] and PDS [43] sum scores which were also directly loading on the GSI. Even though they both asked for major live events, the analysis showed that they independently loading on the GSI and both had an additional influence on the index. In a next step the described structure of the model was improved. By allowing relationships between all latent and manifest variables based on modification indices the statistical adjustments finally led to a fair fitting model. This step showed the importance to consider possible links between all items, factors, and questionnaires [57].

The sample in this study included healthy, middle-aged German soldiers that are regularly examined. This positively affected the extrapolation of the GSI to general population. Regarding to the stress-related questionnaires soldiers showed average values in mean and standard deviation comparable to normative samples [41,46]. Referring to recent findings the PSS4 in our sample was even lower than in the normative sample [58] and the mean of prior stressors was also lower than in a sample of veterans [45]. The similar answer patterns in the questionnaires compared to general population enables the SEM analysis to be extrapolated to findings in the overall population.

### 4.1. Structural Equation Modeling

A beneficial feature of the chosen procedure was the deductive implementation of theory-based questionnaires in the model leading to an inductive examination of the empirical underlying data structure. It is noteworthy that the study was based on a complex statistical method, the SEM analysis. Using SEM, we enabled to examine the links between psychosocial stressors on an item basis by taking the underlying structure of different questionnaires into account. Gallo et al. [59] compiled a variety of separate questionnaires that showed associations between chronic stress burden and prevalence of CHD and stroke. They measured acute stress with the PSS10 and the total number of past and present traumatic events such as natural disaster, combat exposure, and sexual assault; as well as ongoing problems like financial burden, work, or relationship problems. The psychosocial index from Yusuf et al. [8] was developed through a multivariate regression model where all constructs were measured dichotomously. A combination of the psychosocial risk factors with the structural equation modeling method enables the compilation of an individual multidimensional value for stress. In the field of psychosocial stress various studies showed the applicability of using the SEM method for their research (e.g., [60,61]). However, the used content highly depends on the study-context: Ostovar et al. [62] combined stress, anxiety, depression and loneliness to verify the links to internet addiction; Woods-Giscombé and Lobel [63] combined generic stress, race-related stress and gender-related stress in an SEM and developed a multidimensional stress factor. Focussing on CV risk, the combination of specific stress factors is needed as mentioned above [6,64]. The SEM approach has been rarely used to create stress-related indexes in soldiers or in the context of CVD. Another common statistical approach to combine questionnaires is the use of multivariate regression models [8,65]. In comparison to that method, SEM analysis has to take potential causal links or effect chains between variables into consideration which positively affects the precision of a model [66]. Further advantages using the SEM are the following: it takes measurement errors in the observed variables into account, is able to show both direct and indirect effects, enables to develop, estimate, and test complex multivariable models, has a clear conceptualizing of the underlying theory, confirmatory approach and hypothesis testing and is so far the best method for modeling multivariate relations [57,66,67,68]. To our knowledge, the present study is the first attempt to quantify psychosocial risk factors for soldiers as a GSI through SEM.

### 4.2. GSI and Cardiovascular Risk

Apart from the sample and the used statistical method, the range of psychosocial factors and the questionnaire survey method has an important influence on the quality of a stress index which could potentially be used as a MI trigger marker in further research. Many approaches already dealt with the estimation of 10-year CV risk [34,35] as a basis for interventions and risk mitigation. A classical measuring tool for CV-risk is the PROCAM (prospective cardiovascular Münster)-score including already well-established risk factors like gender, age, smoking, systolic blood pressure, and diabetes mellitus [33]. It has recently been shown that also psychosocial stress can trigger MI or cardiac death [69]. It is an independent risk factor for CVD [8] and can increase the risk through moderating biological changes [70]. Consequently, subjective stress measurements should be considered for CV risk and supplementary complement the PROCAM-score as well as other classical risk scores like Framingham [34] or ASCVD [35]. In this context, the standard procedure is that most of the publications focussed on one specific stressor—i.e., earthquake or job stress [71]. However, psychosocial stress is a concept combining different kinds of stress. In the context of CHD specific psychosocial stress factors are closely linked: depressive and anxiety symptoms, negative life events/CM and acute and chronic perceived stress [6,64]. Especially anxiety disorders were found to increase the prevalence of CVD about three-fold [72] and high anxiety levels were associated with the risk of stroke, ventricular arrhythmias, cardiac death, and CVD [73]. It was shown that state-anxiety is associated with higher respiratory sinus arrhythmia magnitude [74]. The same pattern occurred in patients with Generalized Anxiety Disorders linked to lower cardiac vagal control [75] and furthermore higher phobic anxiety was significantly related to ventricular arrhythmias [76]. As underlying mechanism e.g., sympathetic activation, oxidative stress, increased inflammatory mediators and reduced heart rate variability are discussed [73]. Depression or depressive symptoms occur regularly after CV events and are associated with higher mortality rates and increased following CV events [77,78]. In addition, the Takotsubo syndrome should be mentioned here as a specific case of mental stress [79]. It appears from current events like sudden emotional stressors and is known to result in acute heart failure or MI [80]. Anxiety disorders were also found to be significantly more frequent in Takotsubo patients than in patients with an acute coronary syndrome, this association is linked through inflammation leading to sympathetic activity [81]. The long-term mortality consequences are comparable to patients with acute coronary syndrome [82] which underlines the importance to consider mental stress and anxiety in CVD. Some of the physiological and psychological mechanisms of anxiety disorders and the Takotsubo syndrome seem to be comparable therefore both can lead to cardiac arrhythmias or CV events. The choice of using stressful live events as well as the HADS combining anxiety and depressive symptoms among other questionnaires could possibly make the GSI applicable as a screening tool in a wider sense regarding arrhythmia, anxiety, and Takotsubo cardiomyopathy.

A wider range of stressors which then provided more information about the psychosocial aspect as a risk factor was, e.g., collected by Gallo et al. [59]. Amongst PSS10 [42] and the Traumatic Stress Screener [83], the “chronic stress burden” was used, a combination of eight items assessing the number of current ongoing problems. Yusuf et al. [8] and Rosengren et al. [84] expanded the range of aspects in the INTERHEART study and developed a psychosocial index using a multivariate regression model. They created a “general stress scale” that combined the answers for two single questions for stress at home or work and found associations to MI along with depression, locus of control, financial stress, and life events [84].

Following Rosengren et al. [84] and Yusuf et al. [8], our GSI analysis used a similar pattern: perceived acute and chronic stress at work or home was combined to an overall factor while depression, major live events and CM were loading independently on the GSI. In comparison to previous research, the SEM analysis for developing the GSI seems promising to be a future approach in the context of CHD and serves to combine stress questionnaires on an item basis. In our study, only well-established and validated questionnaires were used. To keep the GSI practicable, all single components of work and private stress were measured as a whole by using the questionnaires TICS and PSS4 [41,42] for the assessment of perceived acute and chronic stress.

### 4.3. Limitations of the Study

The exploratory concept of this study was indispensable but further investigations on a different dataset are needed to confirm the structure of the GSI and to increase the extern validity. The sample size was limited and consisted of considerably more male than female soldiers. This distribution reflected the unequal gender distribution in military context, even if 27.1% women in our sample were higher compared to the female proportion in the German army, which was 12.3% in 2019 [85]. These circumstances have to be taken into account when applying our findings to an evenly distributed population. Smaller sample sizes may lead to an underestimated Χ² value, lower RMSEA values and increased standard error of parameter estimation [86,87]. This may have an impact on lower model fit and underestimated significance levels. Furthermore, the multivariate normal distribution was not given in the sample. This is consistent with the Shapiro–Wilk tests showing non-normality for all items. However, studies showed that RMSEA and the parameter estimation are less influenced than the Χ² value by non-normality distribution. As the X²-value was not interpreted in this study the results are expected to be just slightly biased [57,87,88,89]. The missing data in the item set may cause biasing problems when being ignored. The FIML method provided the most efficient unbiased solution of estimating missing variables and has been shown to be superior to listwise or pairwise deletion [90].

## 5. Conclusions

We implemented a GSI to cover mental stress in soldiers through one global factor. In a further step it may enable to focus on psychosocial risk as an additional trigger next to the well-established CVD scores. Therefore, the validation of the GSI is mandatory to prove its suitability for clinical application. The GSI is not just a reflection of the current perception of a person but a holistic index combining information about current stress perception, negative life events, depression, anxiety as well as chronic stress and CM. The GSI globally combines this stress-related information for an easy implementation in clinical practice. All aspects influence each other and play a main role for stress-related mental health and disease.

## Figures and Tables

**Table 1:** Fit indices for all models

Model	Specification	*df*	*Χ*²	RMSEA	AIC	BIC	*Χ*² Test *
Model 1	G-factor	5460	17,615.05 ***	0.108 *** (0.106; 0.109)	51,043.96	51,728.03	
Model 2	One factor for each questionnaire	No result					
Model2a	TICS and PSS4 factor with a factor of higher order	Model not identified					
Model 3	TICS and PSS4 items with perceived stress factor	5457	16,008.86 ***	0.100 *** (0.099; 0.102)	49,443.77	50,137.61	
Model 4	HADS divided in its subscales	5456	16,012.89 ***	0.100 *** (0.099; 0.102)	49,449.80	50,146.90	
Model 4a	HADSA and D with a factor of higher order	Model not identified					
Model 5	Model 3 and CTQ subscales	5452	14,203.58 ***	0.091 *** (0.090; 0.093)	47,648.49	48,358.63	
Model 6	Model 5 and CTQ factor	5451	14,164.89 ***	0.091 *** (0.089; 0.093)	47,611.80	48,325.19	
Model 7	Exclude CTQ_21	5347	13,776.91 ***	0.091 *** (0.089; 0.092)	47,459.57	48,166.44	
Model 8	Intercept: GSI	5346	13,294.53 ***	0.088 *** (0.086; 0.090)	46,979.18	47,689.31	*Χ*²(1) = 482.39 ***
Model 9	Intercept: CTQ_MQ, PSS4_02, PSS4_03	5343	12,560.59 ***	0.084 *** (0.082; 0.086)	46,251.24	46,971.15	*Χ*²(3) = 733.94 ***
Model 10	CTQ_EN~~CTQ_MQ	5342	12,460.37 ***	0.083 *** (0.081; 0.085)	46,153.02	46,876.18	*Χ*²(1) = 100.22 ***
Model 11	TICS_10 ~~ TICS_21 TICS_10 ~~ TICS_41 TICS_10 ~~ TICS_53	5339	12,311.28 ***	0.082 *** (0.081; 0.084)	46,009.93	46,742.87	*Χ*²(3) = 149.09 ***
Model 12	TICS_07 ~~ TICS_22 TICS_07 ~~ TICS_43 TICS_07 ~~ TICS_49	5336	12,182.09 ***	0.082 *** (0.080; 0.084)	45,886.74	46,629.45	*Χ*²(3) = 129.19 ***
Model 13	CTQ_23 ~~ CTQ_24	5335	12,107.42 ***	0.081 *** (0.079; 0.083)	45,814.08	46,560.04	*Χ*²(1) = 74.67 ***
Model 14	TICS_42 ~~ TICS_51	5334	12,003.64 ***	0.081 *** (0.079; 0.083)	45,712.29	46,461.52	*Χ*²(1) = 103.78 ***
Model 15	TICS_22 ~~ TICS_43	5333	11,916.08 ***	0.080 *** (0.078; 0.082)	45,626.73	46,379.21	*Χ*²(1) = 87.56 ***
Model 16	TICS_25 ~~ TICS_36	5332	11,829.89 ***	0.080 *** (0.078; 0.082)	45,542.55	46,298.29	*Χ*²(1) = 86.18 ***
Model 17	CTQ_SA =~ CTQ_25	5331	11,757.61 ***	0.079 *** (0.077; 0.081)	45,472.26	46,231.26	*Χ*²(1) = 158.47 ***

Note. *df* = degrees of freedom; RMSEA = Root Mean Square Error of Approximation; AIC = Akaike’s Information Criteria; BIC = Bayesian Information Criteria; AIC and BIC should only be compared for same item set; ( ) = 90% Confidence Interval; *** = *p* < 0.001; Specification = modifications to the previous model; ~ = loading; ~~ = covariance. PSS4 = Perceived Stress Scale; TICS = Trier Inventory for Chronic Stress; HADS = Hospital Anxiety and Depression Scale; HADSA = HADS Anxiety; HADSD = HADS Depression; CTQ = Childhood Trauma Questionnaire; SA = Sexual Abuse; EN = Emotional Neglect; MQ = minimization questions. Until model 6 theoretical derivations were made. From model 6 onwards, modification indices were taken into account until adequate fit was reached. * *Χ*² test allows model comparisons to the previous model and shows the significance of changes between nested models.

## Data Availability

The data presented in this study are available on request from the corresponding author.

## Appendix A

**Table A1:** Item statistics.

Item	*n*	MIN	MAX	*M*	*SD*	Shapiro–Wilk
TICS_01	192	0	4	2.16	1.12	*W* = 0.91 ***
TICS_02	191	0	4	1.78	0.97	*W* = 0.87 ***
TICS_03	192	0	3	0.85	0.67	*W* = 0.77 ***
TICS_04	191	0	4	1.53	0.98	*W* = 0.90 ***
TICS_05	192	0	4	2.11	0.99	*W* = 0.91 ***
TICS_06	192	0	4	1.56	0.77	*W* = 0.85 ***
TICS_07	192	0	4	2.54	1.14	*W* = 0.89 ***
TICS_08	192	0	4	1.87	0.94	*W* = 0.90 ***
TICS_09	192	0	4	1.57	0.98	*W* = 0.88 ***
TICS_10	192	0	4	1.51	1.09	*W* = 0.89 ***
TICS_11	192	0	4	1.15	1.03	*W* = 0.84 ***
TICS_12	192	0	4	1.68	1.01	*W* = 0.90 ***
TICS_13	192	0	4	1.90	0.87	*W* = 0.87 ***
TICS_14	192	0	4	2.09	0.97	*W* = 0.90 ***
TICS_15	192	0	4	1.11	0.91	*W* = 0.85 ***
TICS_16	192	0	4	1.35	1.05	*W* = 0.88 ***
TICS_17	192	0	4	1.56	1.08	*W* = 0.91 ***
TICS_18	163	0	4	1.28	1.09	*W* = 0.88 ***
TICS_19	192	0	4	1.61	1.03	*W* = 0.90 ***
TICS_20	192	0	4	0.81	0.80	*W* = 0.77 ***
TICS_21	192	0	4	1.47	1.04	*W* = 0.90 ***
TICS_22	192	0	4	2.37	1.03	*W* = 0.90 ***
TICS_23	192	0	4	2.36	1.02	*W* = 0.88 ***
TICS_24	190	0	3	0.63	0.68	*W* = 0.76 ***
TICS_25	192	0	4	1.11	1.01	*W* = 0.86 ***
TICS_26	192	0	4	1.06	0.94	*W* = 0.84 ***
TICS_27	192	0	4	2.26	1.09	*W* = 0.90 ***
TICS_28	192	0	4	1.58	1.05	*W* = 0.90 ***
TICS_29	192	0	4	1.34	0.99	*W* = 0.89 ***
TICS_30	192	0	4	1.72	0.98	*W* = 0.90 ***
TICS_31	192	0	4	1.29	1.09	*W* = 0.89 ***
TICS_32	191	0	4	2.47	0.89	*W* = 0.88 ***
TICS_33	192	0	4	1.01	0.98	*W* = 0.82 ***
TICS_34	191	0	4	0.90	0.96	*W* = 0.83 ***
TICS_35	191	0	4	1.00	0.81	*W* = 0.80 ***
TICS_36	192	0	4	1.25	1.08	*W* = 0.86 ***
TICS_37	192	0	4	1.24	1.00	*W* = 0.88 ***
TICS_38	192	0	4	1.57	1.01	*W* = 0.90 ***
TICS_39	192	0	4	1.80	1.26	*W* = 0.91 ***
TICS_40	192	0	4	1.38	1.05	*W* = 0.89 ***
TICS_41	191	0	4	1.46	1.02	*W* = 0.89 ***
TICS_42	191	0	4	1.14	0.97	*W* = 0.85 ***
TICS_43	191	0	4	2.57	0.92	*W* = 0.89 ***
TICS_44	191	0	4	1.26	0.98	*W* = 0.86 ***
TICS_45	191	0	4	0.76	0.87	*W* = 0.78 ***
TICS_46	190	0	4	1.37	1.11	*W* = 0.88 ***
TICS_47	191	0	4	1.21	0.97	*W* = 0.87 ***
TICS_48	191	0	4	1.63	1.00	*W* = 0.89 ***
TICS_49	190	0	4	2.07	1.19	*W* = 0.91 ***
TICS_50	191	0	4	1.49	1.03	*W* = 0.89 ***
TICS_51	191	0	4	1.15	0.96	*W* = 0.86 ***
TICS_52	191	0	4	0.86	0.85	*W* = 0.81 ***
TICS_53	191	0	4	1.63	0.93	*W* = 0.90 ***
TICS_54	191	0	4	1.09	1.02	*W* = 0.84 ***
TICS_55	191	0	4	0.74	0.78	*W* = 0.79 ***
TICS_56	191	0	4	1.38	1.11	*W* = 0.90 ***
TICS_57	191	0	4	0.97	0.93	*W* = 0.83 ***
PSS4_01	191	0	4	1.63	1.03	*W* = 0.91 ***
PSS4_02	190	0	4	2.87	0.84	*W* = 0.81 ***
PSS4_03	191	0	4	2.56	0.91	*W* = 0.87 ***
PSS4_04	191	0	4	1.23	1.05	*W* = 0.84 ***
CTQ_01	192	1	5	1.18	0.69	*W* = 0.33 ***
CTQ_02	192	1	5	1.63	1.02	*W* = 0.66 ***
CTQ_03	191	1	5	1.53	0.92	*W* = 0.57 ***
CTQ_04	192	1	5	1.17	0.64	*W* = 0.29 ***
CTQ_05	191	1	5	1.79	1.19	*W* = 0.68 ***
CTQ_06	192	1	5	1.09	0.43	*W* = 0.20 ***
CTQ_07	192	1	5	1.76	1.10	*W* = 0.71 ***
CTQ_08	192	1	5	1.29	0.84	*W* = 0.39 ***
CTQ_09	192	1	5	1.22	0.74	*W* = 0.38 ***
CTQ_10	192	1	5	3.22	1.34	*W* = 0.89 ***
CTQ_11	192	1	5	1.32	0.82	*W* = 0.45 ***
CTQ_12	192	1	5	1.44	0.94	*W* = 0.54 ***
CTQ_13	191	1	5	2.04	1.10	*W* = 0.83 ***
CTQ_14	192	1	5	1.77	1.06	*W* = 0.70 ***
CTQ_15	192	1	5	1.19	0.73	*W* = 0.26 ***
CTQ_16	190	1	5	3.38	1.19	*W* = 0.89 ***
CTQ_17	191	1	5	1.13	0.61	*W* = 0.23 ***
CTQ_18	191	1	5	1.32	0.90	*W* = 0.39 ***
CTQ_19	189	1	5	2.20	1.13	*W* = 0.86 ***
CTQ_20	191	1	5	1.16	0.70	*W* = 0.17 ***
CTQ_21	191	1	5	1.05	0.39	*W* = 0.11 ***
CTQ_22	191	1	5	3.50	1.25	*W* = 0.87 ***
CTQ_23	191	1	5	1.14	0.66	*W* = 0.18 ***
CTQ_24	191	1	5	1.13	0.64	*W* = 0.18 ***
CTQ_25	190	1	5	1.21	0.70	*W* = 0.34 ***
CTQ_26	191	1	5	1.40	0.93	*W* = 0.51 ***
CTQ_27	191	1	5	1.11	0.57	*W* = 0.15 ***
CTQ_28	190	1	5	2.22	1.17	*W* = 0.84 ***
HADS_01	192	0	3	0.86	0.71	*W* = 0.80 ***
HADS_02	192	0	3	0.81	0.88	*W* = 0.80 ***
HADS_03	192	0	3	0.74	0.88	*W* = 0.78 ***
HADS_04	191	0	3	0.41	0.63	*W* = 0.68 ***
HADS_05	192	0	3	0.70	0.76	*W* = 0.78 ***
HADS_06	192	0	3	0.45	0.71	*W* = 0.65 ***
HADS_07	192	0	3	0.85	0.79	*W* = 0.82 ***
HADS_08	192	0	3	0.88	0.68	*W* = 0.76 ***
HADS_09	192	0	3	0.52	0.58	*W* = 0.69 ***
HADS_10	191	0	3	0.43	0.71	*W* = 0.67 ***
HADS_11	192	0	3	0.98	0.79	*W* = 0.83 ***
HADS_12	192	0	3	0.54	0.72	*W* = 0.74 ***
HADS_13	192	0	2	0.31	0.56	*W* = 0.58 ***
HADS_14	192	0	3	0.40	0.72	*W* = 0.59 ***
DRRI_2	178	0	9	2.04	2.10	*W* = 0.83 ***
PDS	163	0	5	1.80	1.45	*W* = 0.92 ***

Note: *N* = sample size; *MIN* = minimum; *MAX* = maximum; *M* = arithmetic mean; *SD* = standard deviation; *W* = Shapiro–Wilk test statistic; PSS4 = Perceived Stress Scale; TICS = Trier Inventory for Chronic Stress; HADS = Hospital Anxiety and Depression Scale; PDS = Posttraumatic Stress Diagnostic Scale Checklist; DRRI_2 = Deployment Risk and Resilience Inventory; CTQ = Childhood Trauma Questionnaire; *** = *p* < 0.001; higher values indicate more negative outcomes.

## Appendix B

**Table A2:** Additional statistics and results for Model 17: loadings, variances, and covariances.

Items/Factors		Loading/Covariance/Intercept	*SE* Loading	*p*	Variance	*SE* Variance	*p*
*GSI ~*					0.31	0.05	<0.001
	1	2.10	0.09	<0.001			
	*perceived stress*	1.00			0.24	0.05	<0.001
	*HADS*	0.40	0.03	<0.001	0.17	0.03	<0.001
	*CTQ*	0.71	0.04	<0.001	0.15	0.03	<0.001
	PDS	0.85	0.06	<0.001	1.98	0.23	<0.001
	DRRI_2	1.02	0.08	<0.001	3.73	0.41	<0.001
*perceived stress~*							
	TICS_01	1.00			0.94	0.10	<0.001
	TICS_02	0.84	0.04	<0.001	0.59	0.06	<0.001
	TICS_03	0.41	0.02	<0.001	0.33	0.03	<0.001
	TICS_04	0.74	0.03	<0.001	0.60	0.06	<0.001
	TICS_05	0.95	0.04	<0.001	0.92	0.10	<0.001
	TICS_06	0.72	0.03	<0.001	0.42	0.04	<0.001
	TICS_07	1.12	0.05	<0.001	1.26	0.12	<0.001
	TICS_08	0.85	0.04	<0.001	0.81	0.09	<0.001
	TICS_09	0.76	0.03	<0.001	0.59	0.06	<0.001
	TICS_10	0.67	0.04	<0.001	0.89	0.08	<0.001
	TICS_11	0.55	0.04	<0.001	0.89	0.09	<0.001
	TICS_12	0.79	0.04	<0.001	0.74	0.08	<0.001
	TICS_13	0.88	0.04	<0.001	0.55	0.06	<0.001
	TICS_14	0.95	0.04	<0.001	0.82	0.09	<0.001
	TICS_15	0.54	0.03	<0.001	0.61	0.06	<0.001
	TICS_16	0.54	0.03	<0.001	0.74	0.08	<0.001
	TICS_17	0.76	0.04	<0.001	0.75	0.08	<0.001
	TICS_18	0.63	0.04	<0.001	0.84	0.09	<0.001
	TICS_19	0.78	0.04	<0.001	0.60	0.06	<0.001
	TICS_20	0.41	0.03	<0.001	0.47	0.05	<0.001
	TICS_21	0.67	0.04	<0.001	1.04	0.11	<0.001
	TICS_22	1.06	0.05	<0.001	1.06	0.11	<0.001
	TICS_23	1.06	0.05	<0.001	1.01	0.11	<0.001
	TICS_24	0.33	0.02	<0.001	0.32	0.03	<0.001
	TICS_25	0.57	0.03	<0.001	0.61	0.06	<0.001
	TICS_26	0.52	0.03	<0.001	0.65	0.07	<0.001
	TICS_27	1.05	0.05	<0.001	0.87	0.09	<0.001
	TICS_28	0.77	0.04	<0.001	0.64	0.07	<0.001
	TICS_29	0.65	0.03	<0.001	0.71	0.07	<0.001
	TICS_30	0.78	0.04	<0.001	0.94	0.01	<0.001
	TICS_31	0.64	0.04	<0.001	0.81	0.08	<0.001
	TICS_32	1.10	0.05	<0.001	0.89	0.10	<0.001
	TICS_33	0.50	0.03	<0.001	0.71	0.07	<0.001
	TICS_34	0.46	0.03	<0.001	0.70	0.07	<0.001
	TICS_35	0.49	0.03	<0.001	0.48	0.05	<0.001
	TICS_36	0.63	0.04	<0.001	0.78	0.08	<0.001
	TICS_37	0.61	0.03	<0.001	0.68	0.07	<0.001
	TICS_38	0.78	0.03	<0.001	0.46	0.05	<0.001
	TICS_39	0.86	0.05	<0.001	1.20	0.12	<0.001
	TICS_40	0.67	0.04	<0.001	0.74	0.08	<0.001
	TICS_41	0.62	0.04	<0.001	1.25	0.13	<0.001
	TICS_42	0.55	0.03	<0.001	0.77	0.08	<0.001
	TICS_43	1.15	0.05	<0.001	0.84	0.09	<0.001
	TICS_44	0.64	0.03	<0.001	0.49	0.05	<0.001
	TICS_45	0.41	0.03	<0.001	0.53	0.05	<0.001
	TICS_46	0.68	0.04	<0.001	0.81	0.08	<0.001
	TICS_47	0.60	0.03	<0.001	0.62	0.07	<0.001
	TICS_48	0.74	0.04	<0.001	0.94	0.10	<0.001
	TICS_49	0.94	0.05	<0.001	1.26	0.13	<0.001
	TICS_50	0.73	0.03	<0.001	0.62	0.07	<0.001
	TICS_51	0.56	0.03	<0.001	0.67	0.07	<0.001
	TICS_52	0.43	0.03	<0.001	0.52	0.05	<0.001
	TICS_53	0.74	0.04	<0.001	0.78	0.08	<0.001
	TICS_54	0.57	0.03	<0.001	0.62	0.07	<0.001
	TICS_55	0.38	0.02	<0.001	0.41	0.04	<0.001
	TICS_56	0.66	0.04	<0.001	0.98	0.10	<0.001
	TICS_57	0.50	0.03	<0.001	0.56	0.06	<0.001
	PSS4_01	0.79	0.04	<0.001	0.65	0.07	<0.001
	PSS4_02	−0.42	0.08	<0.001	0.60	0.06	<0.001
	PSS4_03	−0.45	0.09	<0.001	0.71	0.07	<0.001
	PSS4_04	0.61	0.03	<0.001	0.74	0.08	<0.001
*HADS~*							
	HADS_01	1.00			0.30	0.04	<0.001
	HADS_02	1.01	0.07	<0.001	0.47	0.05	<0.001
	HADS_03	0.96	0.07	<0.001	0.46	0.05	<0.001
	HADS_04	0.59	0.05	<0.001	0.24	0.03	<0.001
	HADS_05	0.89	0.06	<0.001	0.31	0.04	<0.001
	HADS_06	0.63	0.05	<0.001	0.33	0.04	<0.001
	HADS_07	1.02	0.06	<0.001	0.39	0.04	<0.001
	HADS_08	0.95	0.06	<0.001	0.38	0.04	<0.001
	HADS_09	0.61	0.05	<0.001	0.25	0.03	<0.001
	HADS_10	0.56	0.05	<0.001	0.40	0.04	<0.001
	HADS_11	1.03	0.07	<0.001	0.59	0.06	<0.001
	HADS_12	0.73	0.05	<0.001	0.32	0.03	<0.001
	HADS_13	0.44	0.04	<0.001	0.23	0.02	<0.001
	HADS_14	0.56	0.05	<0.001	0.38	0.04	<0.001
*CTQ~*							
	*CTQ_EA*	1.00			0.14	0.03	<0.001
	*CTQ_SA*	0.77	0.04	<0.001	0.28	0.03	<0.001
	*CTQ_PA*	0.81	0.04	<0.001	0.12	0.02	<0.001
	*CTQ_EN*	1.19	0.07	<0.001	0.26	0.04	<0.001
	*CTQ_PN*	0.76	0.04	<0.001	0.04	0.01	<0.001
	*CTQ_MQ*	−1.20	0.11	<0.001	0.59	0.09	<0.001
*CTQ_EA~*							
	CTQ_03	1.00			0.56	0.06	<0.001
	CTQ_08	0.89	0.04	<0.001	0.29	0.04	<0.001
	CTQ_14	1.19	0.05	<0.001	0.52	0.07	<0.001
	CTQ_18	0.94	0.04	<0.001	0.24	0.03	<0.001
	CTQ_25	0.34	0.05	<0.001	0.22	0.02	<0.001
*CTQ_SA~*							
	CTQ_20	1.00			0.08	0.01	<0.001
	CTQ_23	0.97	0.02	<0.001	0.08	0.01	<0.001
	CTQ_24	0.96	0.02	<0.001	0.06	0.01	<0.001
	CTQ_27	0.94	0.02	<0.001	0.00	0.00	0.427
*CTQ_PA~*							
	CTQ_09	1.00			0.27	0.03	<0.001
	CTQ_11	1.11	0.04	<0.001	0.20	0.03	<0.001
	CTQ_12	1.18	0.05	<0.001	0.51	0.06	<0.001
	CTQ_15	0.99	0.04	<0.001	0.21	0.03	<0.001
	CTQ_17	0.93	0.03	<0.001	0.10	0.02	<0.001
*CTQ_EN~*							
	CTQ_05	1.00			0.81	0.09	<0.001
	CTQ_07	1.01	0.04	<0.001	0.43	0.05	<0.001
	CTQ_13	1.14	0.05	<0.001	0.45	0.05	<0.001
	CTQ_19	1.24	0.05	<0.001	0.36	0.05	<0.001
	CTQ_28	1.26	0.05	<0.001	0.26	0.04	<0.001
*CTQ_PN~*							
	CTQ_01	1.00			0.40	0.05	<0.001
	CTQ_02	1.46	0.07	<0.001	0.57	0.07	<0.001
	CTQ_04	1.02	0.05	<0.001	0.26	0.03	<0.001
	CTQ_06	0.93	0.04	<0.001	0.11	0.02	<0.001
	CTQ_26	1.25	0.07	<0.001	0.53	0.06	<0.001
*CTQ_MQ~*							
	1	4.98	0.18	<0.001			
	CTQ_10	1.00			0.95	0.11	<0.001
	CTQ_16	1.06	0.03	<0.001	0.35	0.05	<0.001
	CTQ_22	1.10	0.03	<0.001	0.26	0.04	<0.001
PSS4_02	1	3.50	0.19	<0.001			
PSS4_03	1	3.76	0.17	<0.001			
*CTQ_SA~*	CTQ_25	0.57	0.06	<0.001			
*CTQ_EN~~*	*CTQ_MQ*	−0.33	0.05	<0.001			
CTQ_23~~	CTQ_24	0.05	0.01	<0.001			
TICS_10~~							
	TICS_21	0.27	0.08	0.001			
	TICS_41	0.48	0.09	<0.001			
	TICS_53	0.24	0.06	<0.001			
TICS_07~~							
	TICS_22	0.53	0.09	<0.001			
	TICS_43	0.53	0.09	<0.001			
	TICS_49	0.43	0.10	<0.001			
TICS_42~~	TICS_51	0.47	0.06	<0.001			
TICS_22~~	TICS_43	0.58	0.08	<0.001			
TICS_25~~	TICS_36	0.42	0.06	<0.001			

Note: *SE* = Standard Error; *p* = *p*-value; ~ = loading; ~~ = covariance; Variables in normal type are items; words in Italic type are factors. Loadings for variables with a 1 in the third column were restricted to 1 or show intercepts. PSS4 = Perceived Stress Scale; TICS = Trier Inventory for Chronic Stress; HADS = Hospital Anxiety and Depression Scale; PDS = Posttraumatic Stress Diagnostic Scale Checklist; DRRI_2 = Deployment Risk and Resilience Inventory; CTQ = Childhood Trauma Questionnaire; EA = Emotional Abuse; PA = Physical Abuse; SA = Sexual Abuse; EN = Emotional Neglect; PN = Physical Neglect; MQ = minimization questions.
